# How to optimise drug study design: pharmacokinetics and pharmacodynamics studies introduced to paediatricians

**DOI:** 10.1111/jphp.12637

**Published:** 2016-09-27

**Authors:** Eric Vermeulen, John N. van den Anker, Oscar Della Pasqua, Kalle Hoppu, Johanna H. van der Lee, Global Research in Paediatrics (GRiP)

**Affiliations:** ^1^ Pediatric Clinical Research Office Emma Children's Hospital Academic Medical Center Amsterdam The Netherlands; ^2^ Division of Pediatric Clinical Pharmacology Children's National Health System Washington DC USA; ^3^ Division of Paediatric Pharmacology and Pharmacometrics University of Basel Children's Hospital Basel Switzerland; ^4^ Intensive Care and Department of Pediatric Surgery Erasmus Medical Center Sophia Children's Hospital Rotterdam The Netherlands; ^5^ Clinical Pharmacology Modelling & Simulation GlaxoSmithKline Stockley Park UK; ^6^ Clinical Pharmacology & Therapeutics University College London London UK; ^7^ Poison Information Centre Helsinki University Central Hospital Helsinki Finland

**Keywords:** paediatrics, pharmacodynamics, pharmacokinetics

## Abstract

**Objectives:**

In children, there is often lack of sufficient information concerning the pharmacokinetics (PK) and pharmacodynamics (PD) of a study drug to support dose selection and effective evaluation of efficacy in a randomised clinical trial (RCT). Therefore, one should consider the relevance of relatively small PKPD studies, which can provide the appropriate data to optimise the design of an RCT.

**Methods:**

Based on the experience of experts collaborating in the EU‐funded Global Research in Paediatrics consortium, we aimed to inform clinician‐scientists working with children on the design of investigator‐initiated PKPD studies.

**Key findings:**

The importance of the identification of an optimal dose for the paediatric population is explained, followed by the differences and similarities of dose‐ranging and efficacy studies. The input of clinical pharmacologists with modelling expertise is essential for an efficient dose‐finding study.

**Conclusions:**

The emergence of new laboratory techniques and statistical tools allows for the collection and analysis of sparse and unbalanced data, enabling the implementation of (observational) PKPD studies in the paediatric clinic. Understanding of the principles and methods discussed in this study is essential to improve the quality of paediatric PKPD investigations, and to prevent the conduct of paediatric RCTs that fail because of inadequate dosing.

It is unfortunate that a communication gap still exists between paediatricians and clinical pharmacologists, who can apply methodologies to validate current prescription practice, in many cases without the need for additional prospective trials.^1^

## Introduction

Children have traditionally been protected from participation in medical (drug) research, and fas a consequence, medications have not been appropriately labelled for them.^2^ Regulatory initiatives such as the Paediatric Research Equity Act and Best Pharmaceuticals for Children's Act in the United States and the Paediatric Regulation in the European Union (EU) provide incentives for pharmaceutical companies to investigate *new* drugs in children. Sponsors can submit a paediatric investigation plan to support the authorisation of a new drug for children.^3^ However, off‐label dosing recommendations for currently marketed drugs need to be revisited,^1^, ^4^, ^5^, ^6^, ^7^, ^8^ especially for older, off‐patent medications.^7^ Given the general lack of interest in the ‘paediatric‐use marketing authorisation’ opportunity, which provides sponsors incentives for research on off‐patent drugs, the initiative to gather empirical evidence to support the dose rationale for older drugs is left to non‐commercial (academic) paediatric clinician‐scientists.^9^ In fact, the need for increasing awareness of paediatricians about the value of investigator‐initiated trials in children is acknowledged in the revision of Directive of the European Commission (EC) in 2014, which tries to correct the bias towards trials sponsored by pharmaceutical companies, ‘while those with non‐commercial sponsors were overlooked’.^10^, ^11^ Another element that has been highlighted in the revised directive is the role of paediatric networks to help consolidate available knowledge about medicines and translate it into practice.^12^, ^13^ To meet the demand for clinical trials, ‘the pediatric research enterprise must act with diligence to address deficiencies in our current preclinical and clinical research systems that often give rise to irreproducible data. Historically, most federally funded pediatric research programs were designed to generate data for publication rather than regulatory review, the latter a standard that needs to withstand independent validation down to individual elements’.^13^ Paediatric drug research poses challenges, but innovations in trial design and pharmacology prompt Rieder and Hawcutt^14^ to conclude that ‘there has never been a better time for conducting drug studies in children’.

The general principles of randomised clinical trials (RCTs) to study drug efficacy and effectiveness are well known among most paediatricians. However, they may be unaware that other types of studies, that is studies to identify the appropriate dose and dosing regimen in children, might have a higher priority on the research agenda. Failure to perform these studies can lead to a negative trial result, not because of insufficient statistical power (type II error), but because of inadequate dose selection; that is, the drug dose that is compared to placebo or another comparator results in too low exposure to ensure the required clinical response in children. This was illustrated by a retrospective investigation of the design aspects that might have caused the failure of several antihypertensive dose–response trials submitted to the Food and Drug Administration from 1998 to 2005.^15^ The authors recommend that ‘future pediatric antihypertensive trials should incorporate a wide range of doses and use information from adult trials to account for potential pharmacological differences between adult and pediatric populations’. As long as there are no safety concerns, for dose–response trials these authors advise to use a lowest dose that is lower than the lowest approved relative dose (per kg or per m^2^) in adults, and a highest relative dose that is at least twofold higher than the highest approved relative dose in adults. We would be more cautious and more specific about how to evaluate a medicine, but we agree that characterisation of the exposure–response curve requires the evaluation of dose levels that result in a wide range of drug exposure, including in some cases nominal dose levels that may be lower or higher than the currently approved therapeutic doses in adults.

This study aimed to close the communication gap between clinical pharmacologists and paediatricians and provide a starting point for the design of paediatric dose‐finding studies in such a way that the results can be used to justify the dose rationale for children and consequently to support the development of clinical guidelines and labelling changes. We want to make clear (1) why the identification of an optimal dose for the paediatric population is important, (2) what the differences and similarities are in the design and conduct between dose‐ranging and efficacy studies and (3) which information is needed for the planning of a dose‐finding study and how this can be obtained.

### Why the identification of an optimal dose for the paediatric population is important

Many drugs used in daily paediatric practice lack a scientifically sound, evidence‐based dosing regimen.^16^, ^17^ Off‐label doses in children are often the result of an extrapolation exercise; that is, they are based on the adult dose corrected only for differences in body size (e.g. body weight or body surface area (BSA)). Such extrapolations often rely on the assumption of a linear correlation between dose and size. In fact, when using doses per kg or per square metre, one implicitly assumes that fractioning of the dose will result in comparable drug levels; that is, concentrations change in a linear fashion with weight or BSA, respectively. This practice also assumes that children and adults are comparable with regard to body composition and have similar gastrointestinal, renal and hepatic function (primary organs determining the absorption, distribution and metabolism of drugs), as well as concentration–response relationships. As developmental changes are mostly nonlinear, this so‐called empirical dosing can lead to over‐ or underdosing, especially in specific age groups such as neonates and (extremely) low‐birthweight infants, thereby increasing the risk of toxicity or reduced efficacy. The heterogeneity within the paediatric population, ranging from very small premature neonates to, sometimes overweight or obese, 18‐year‐olds, cannot be overemphasised.

To ensure that the aforementioned points are considered for the selection of the dose and design of a clinical study, a few basic concepts should be highlighted. Pharmacokinetics (PK) describes what happens to a drug when it enters the body (including absorption, distribution, metabolism and excretion), and pharmacodynamics (PD) refers to the effect the drug has on the body. Historically, a major constraint for the evaluation of the dose rationale has been the lack of information about drug exposure. Traditional PK studies involve the collection of multiple blood samples in each patient, usually taken according to a rigidly timed and structured protocol, within a relatively small patient population (e.g. *n* = 12). This ‘data‐rich’ approach has severe limitations in paediatric practice for both ethical and practical reasons: the fixed sampling strategy potentially interferes with patient care; and the requirement for multiple blood samples (perhaps 12–15) raises concerns about venous access and blood loss. Population PK (using sparse sampling schemes in which less blood samples are taken per individual without the need for a rigid sampling time as compared to classical PK studies) and PKPD modelling (using statistical models to characterise the exposure–response relationship of a drug) are now well established.^18^, ^19^, ^20^, ^21^, ^22^, ^23^ This approach prevents children being exposed to the practice of large numbers and volumes of blood sampling seen in adult PK and PKPD studies.

Whereas the conduct of a PK study may suffice to support the dose rationale in some cases (e.g. when evidence exists of comparable exposure–response relationships in adults and children), clinicians and investigators are less familiar with the requirements and conditions in which a PKPD study is necessary. The criteria were initially set out in a regulatory guidance, in which the FDA proposed a ‘paediatric study decision tree’.^24^ This diagram shows the requirements for using adult data (or any other reference group or population) to extrapolate or infer efficacy and safety in (specific groups of) children. Evidence that disease progression, PKPD relationships and endpoints are similar or comparable in both adults and children allows the use of PK (bridging) studies to support the dose rationale for the paediatric population. However, if these requirements are not met, the decision tree clearly indicates the need for further PKPD or efficacy studies. It is important to understand that regulatory views in the European Union are slightly different from those in the United States. According to a reflection paper released by the European Medicines Agency (EMA), extrapolation may be generally defined as: ‘Extending information and conclusions available from studies in one or more subgroups of the patient population (source population), or in related conditions or with related medicinal products, to make inferences for another subgroup of the population (target population), or condition or product, thus reducing the need to generate additional information (types of studies, design modifications, number of patients required) to reach conclusions for the target population, or condition or medicinal product’. (EMA 25: 2) Instead of a decision tree, the European regulators propose a framework to systematically determine whether extrapolation can be applied, introducing the requirement for an extrapolation plan and what such a plan should entail.^26^

The creation of a framework for extrapolations has also made explicit which are the requirements for data generation, in particular how studies should be designed following the extrapolation plan, including the relevance of PKPD and dose‐ranging studies. The extrapolation plan represents therefore a mechanism to ensure the accurate use of current knowledge as well as the criteria for the use of biomarkers and clinical endpoints, many of which have not been evaluated or qualified to support a regulatory application. An example of a study that has led to incorporation of the starting dose and titration scheme (of argatroban) in the US prescribing information is a study by Madabushi *et al*.^27^ An example of the use of a PD endpoint that has been validated for use in children is the measurement of pain in young children in De Cock *et al*.^28^

As these types of study have been an area of expertise within pharmaceutical R&D, academic investigators still have limited experience with their implementation. It should therefore be clear that before performing a RCT, the doses to be tested need to be selected and justified; otherwise, trials may fail as has happened in the past.^15^ Most importantly, paediatricians need to understand that body size (weight) is not necessarily a surrogate or proxy for differences in physiological or organ function across the various subgroups of the paediatric population. During the planning and evaluation of the suitable dose(s) and dosing regimens for children, different factors may need to be considered in an integrated manner, taking into account differences (as compared to adults) due to demographic and clinical factors as well as the role of organ maturation, ontogeny of enzymes and developmental growth.^29^

It is also worth mentioning that whereas maturation and ontogeny play a critical role in very young children (e.g. preterm newborns, term newborns, infants, toddlers), the use of postnatal or even postmenstrual age does not necessarily provide insight into organ function at an individual patient level. For instance, one can use postmenstrual age to refer to the average (patho)physiological difference in glomerular filtration in preterm newborns, but one should measure cystatin C to obtain accurate estimates of the organ function in a given patient. In other words, the use of age as a proxy or surrogate for function is of limited value, given the large heterogeneity in organ maturation.^30^ Given the wide weight variation (see, e.g., quartiles of the weight by age growth curves for male and female patients from the World Health Organization and National Center for Health Statistics^31^, ^32^), the use of age as criterion for dosing medicines in older children yields even larger errors. Similarly, there is little scientific basis to support the use of dosing based on BSA, as BSA does not accurately reflect differences in organ or metabolic function. ‘Scaling for function’ is suggested^1^ in which the dosing accounts for developmental growth and different (patho)physiological conditions.^24^ BSA was introduced as a correction factor for dosing regimens associated with poor tolerability, and dates back to the introduction of cytotoxic medicines in oncology. Current understanding of drug disposition and PKPD relationships strongly suggests that weight‐ or biomarker‐banded dosing regimens should be used if large heterogeneity is anticipated in a given group of patients or disease condition (e.g. renally impaired patients).

### Differences and similarities in the design and conduct between dose‐finding and efficacy studies

Dose‐ranging studies, also known as phase II studies, occupy a key position in clinical drug development. If properly designed and accurately performed, a dose‐finding study will save time and effort during the assessment of efficacy in comparative and large‐scale trials in phase III. Moreover, evidence from these studies may help to minimise the numbers of patients required in subsequent phases of development or even eliminate the need for additional data.^33^

A key goal of phase II is to determine the effective dose(s) that will inform a phase III trial. Often the results of phase II studies will substantiate the dose and dosing regimen that will be used on the product label submitted for approval as part of the new drug application. Whereas current regulatory guidelines highlight the importance of identifying an effective and safe dose as the basis for approval of a novel medicine, an overwhelming number of examples show that the characterisation of the exposure–response curve and subsequent selection of the optimal dose range can have important implications for the development of the medicinal product.^34^ An optimal dose is a dose that is high enough to demonstrate efficacy in the target population taking into account the impact of variability in pharmacokinetics and pharmacodynamics. Yet, this dose should ensure minimum safety concerns and adverse events. There are different strategies or approaches to determine the optimal dose, and the three most common dose‐finding study designs are described below.

#### Parallel dose comparison

Parallel dose comparison studies are the classical dose‐finding studies.^35^ This is still one of the most common (but also the least efficient) study designs. In a parallel dose comparison study, several potential doses are selected and patients are randomised to receive one of the doses or placebo for the entire study period. At the end of the study, the outcome in each treatment group is compared to the placebo group. Given that these designs are not staggered, all treatment groups, including the higher dose cohorts, may be evaluated in parallel. Therefore, this study design is best suited for situations where there is some confidence about the location of the exposure–response curve and no concern about the safety profile of the compound. On the other hand, parallel dose comparisons are very inefficient designs. They can make the identification of the optimal dose and dosing regimen rather challenging if limited information is available about the location of the dose–response curve. Empirical choice of the doses to be used in a (paediatric) study may lead to biased estimates of the parameters describing the dose–response curve. Dose‐finding parallel group studies are difficult to perform in children due to the relatively narrow dose range, the small interval between tested doses, the interindividual variability of the parameters measured and therefore the lack of statistical power. The ‘continual reassessment method’ has been used in several instances in children. This method allocates doses sequential to groups of patients. The first group is treated with the first dose level, whereas dose levels for the subsequent groups are determined according to the model estimates of the dose–efficacy and dose–safety relationships.^36^, ^37^ The implications of traditional approaches vs model‐based data analysis for antidepressant drugs were evaluated by Santen *et al*.^38^, ^39^

#### Staggered dose escalation

If there is uncertainty about the safety profile of a medicinal product, one can start exposing patients to lower doses first before progressing to higher doses. In this type of study, one starts with one group of patients (often referred to as a cohort) and assigns them to a low‐dose treatment, during which the group is observed for some period of time. If no safety issues are encountered, a new group of patients can be enrolled and assigned to a higher dose. This process is repeated until the clinical response is achieved or the maximum tolerated dose is reached. This design increases patient safety because you can start by exposing a small number of patients to the lowest dose possible, which might discriminate drug response from baseline or control treatment. By doing so, one mitigates risk by limiting both the initial number of patients and the exposure of each patient to study drug. As indicated above, control participants can be included along with each cohort if the objective is to compare efficacy with standard of care or other reference treatment.^33^

#### Intrapatient dose titration

In a dose titration study, titration is aimed at achieving a predefined clinical response or maximum tolerated dose within a patient. This means that each patient will start at a low dose and receive an incrementally higher dose until a predefined clinical response or maximum tolerated dose is reached. Dose titration studies work well in chronic conditions where a drug will be used for a long period of time, and where it is likely that significant differences will be seen in the way each patient reacts. Epilepsy is a good example of a condition where dose titration is useful.^40^ There is considerable variability in how individual patients respond to antiepileptic products, and with titrating the dose, one can tailor treatment with lower doses to patients who are more responsive to treatment and higher doses to those who do not respond optimally to the same dose level.

Whereas mainstream data analysis in efficacy trials in adults relies primarily on treatment comparisons, as assessed by hypothesis testing (e.g. ANOVA), paediatric dose‐finding studies can benefit enormously from a model‐based approach, in which treatment effects are not estimated primarily based on pairwise comparisons, but by PKPD parameter estimates. Among the many advantages, PKPD modelling^19^ of dose‐finding data allows effective separation of the variability in response associated with differences in drug exposure from other factors known to cause variation in response. Moreover, data analysis can be complemented by simulations, including scenarios which expand the population characteristics to include characteristics of virtual participants who were not included in the empirical study, providing insight into the implications of the dose and response across the overall target population.

Another potential benefit of the use of model‐based approaches (using statistical models for predicting the effect and efficacy of a drug) is the possibility of eliminating the need for additional data, thereby avoiding the exposure of children to unnecessary experimental protocol procedures. In contrast to traditional (descriptive) experimental protocols, the use of modelling does not limit to summarising the experimental variables. It relies on the estimation of parameters, which describe either the disposition (e.g. clearance, distribution volume) or PKPD relationships (e.g. potency) as the basis for extrapolation and prediction of drug exposure and response in a new patient or group of patients, taking into account individual characteristics and variability in drug PK or PD parameters. Given that assumptions can be made about the magnitude of the changes associated with growth and maturation, mathematical functions exist that allow for scaling of model parameters. For instance, volume of distribution and clearance are known to change with body weight. Using allometric scaling, it is possible to predict how volume decreases as body weight becomes smaller. Examples where adult data have been used to support paediatric dose selection include the work performed by Avramis *et al*.^41^ and Piana *et al*.^23^

In addition, population PK and PKPD models allow for the identification of additional covariate effects, including demographic and clinical factors, such as creatinine clearance. Evidence of the influence of such covariates on PK or PKPD relationships can be used to predict the impact of overall variability on drug exposure and treatment response. Most importantly, the parameter estimates obtained by extrapolation can be directly used as the basis for dosing recommendations.^42^, ^43^

One can also characterise the effect of demographic and clinical factors on pharmacokinetics and discriminate them from factors that influence the variability in pharmacodynamics, for example disease severity or baseline conditions. This stepwise approach is often referred to in specialised literature as hierarchical modelling and has the main advantage of describing both identifiable and non‐identifiable sources of variability. Each ‘variability’ component is expressed in a hierarchical model as a different parameter. Identifiable sources of variability are converted into covariate factors during the analysis, whereas non‐identifiable sources are expressed as statistical distributions. Variability, in this context, is typically split into between‐patient variability, between‐occasion variability (within the same patient on different occasions during the course of treatment) and residual variability in the measurements.^22^ The implementation of this type of analysis can be performed using different techniques and software programs. The most commonly used software for population PK and PKPD modelling is NONMEM (Icon Development Plc, South County Business Park, Leopardstown Dublin 18, Ireland). However, other tools exist that can be used to support the development of nonlinear mixed‐effects modelling including, for example, SAS, Monolix, USC*PAC, MATLAB and ADAPT.^44^, ^45^

In addition to the advantages relative to the methodological aspects described above, the use of a model‐based approach allows one to take into account additional challenges that are faced when collecting and interpreting paediatric data. For instance, it is possible to consider a more mechanistic approach through incorporation of physiologically based pharmacokinetic models, which are able to factor in the contribution of maturation processes in drug disposition in very young children.

In the era of evidence‐based medicine, RCTs remain the best known approach for the evaluation of efficacy. The main difference between PKPD studies or dose‐finding studies and randomised efficacy trials is the type of information that is generated and the objective of the study. In a typical RCT, the main objective is to establish the statistical significance of the mean difference in outcomes between the intervention groups. The entire study design is aimed at minimising variability or ‘noise’ around this ‘signal’. In a PKPD study, on the other hand, the main objective is to establish how response changes with varying exposure and whenever possible identify the causes or sources of within‐ and between‐patient variability. In this respect, patient characteristics such as age, renal function, maturation status and disease severity can all play an important role and lead to biased estimates of the exposure–response curve, if not adjusted for. Basically, this difference can be observed as a variation on the distinction in two (psychological) scientific paradigms that were described by Cronbach in 1957,^46^ that is (1) the correlational approach, in which the investigator uses variation between patients to study the correlation with the determinants of this variation, and (2) the experimental approach, where the investigator attempts to measure change due to an intervention (the signal) with as much precision (as little noise) as possible.

The ‘learning‐confirming’ paradigm proposed by Sheiner,^47^ which has been acknowledged by the FDA as an important step to establish exposure–response and support dose rationale, enables optimisation of the process to learn about exposure–response relationship if knowledge cannot be extrapolated from adult studies.

### Information needed for the planning of a dose‐finding study, and how this can be obtained

The following provides basic information on the elements that should be considered when planning a dose‐finding study. We want to emphasise that the first step when planning such a study is to consult all the important players: clinicians, nurses, patients/parents, pharmacists, geneticists and clinical pharmacologists with modelling expertise. Obviously, the exact composition of the team will depend on the investigational product. The clinical pharmacologist can advise on the design of the study and minimisation of patient samples. The GRiP initiative offers an educational programme for paediatric investigators interested in this type of research.^48^

One of the consequences of the difference between typical RCTs for the evaluation of efficacy and PKPD studies is the different emphasis, that is from statistical power and sample size for hypothesis testing to parameter accuracy and precision for model fitting. The precision of PK and exposure–response parameters is critical in the sample size calculation for paediatric PKPD studies. Prior knowledge of the disease, exposure, and response from adults and other relevant paediatric data, such as that related to variability, can be used to derive the optimal sample size for ensuring precise parameter estimation. The investigators should account for all potential sources of variability, including interpatient and intrapatient variability, and differences between the adult and paediatric populations in the final selection of the sample size for each age group. Simulations can play a key role in that process, as variability is not considered to be only random. Moreover, it is the evidence of an exposure–response relationship that should define the success of the trial, not the statistical significance of eventual differences between treatment arms.

The distinct age groups to be studied should be chosen based upon what is known about the prevalence and incidence of the disease, taking into account the role of developmental growth, maturation processes and ontogeny, all of which can affect pharmacokinetics, pharmacodynamics and the safety profile of a drug.

If the drug is intended for use in newborn infants, the paediatric study plan should specify whether premature or small‐for‐gestational‐age infants will be included in the study population. Given the influence of different factors on pharmacokinetic and pharmacodynamic variability, it is important to ensure all relevant information is captured for each patient, for instance gestational age and serum creatinine or cystatin C for preterm infants, birthweight and actual weight for infants and toddlers.

In 2012, the FDA discussed a proposal, ultimately rejected by the Advisory Committee, for a sample size standard for paediatric pharmacokinetic studies, which stated that a study had to be powered with at least 80% to target a confidence interval with no more than 20% relative standard error in the pharmacokinetic parameter estimates,^49^ but with nonlinear mixed‐effects methods, also known as population approach, sample size is not the only relevant aspect. Sample size calculations are well explained by Roberts *et al*.,^50^ who also describe the software programs available for this purpose. Although these authors show that for every situation an ‘optimal’ sample size and study design can and should be determined, they seem to overlook important feasibility issues that need to be considered, especially when dealing with newborns and toddlers. Important for paediatricians is that PKPD studies do not necessarily follow the same design route as classical RCTs. PKPD studies are designed with the objective of learning about the appropriate dose, and hence must not follow the logic of the classical study that aims to determine the difference in outcome between groups.

Non‐compartmental analysis based on rich PK sampling has been common practice for a large number of paediatric trials. The use of frequent blood sampling has led to important ethical and practical challenges in the implementation of clinical trials. This situation can be improved by better understanding of paediatricians about the value of model‐based approaches. Population PK and PKPD modelling analysis based on sparse PK sampling can achieve sufficient precision for the characterisation of PK and PKPD parameters.^49^

From the above, it is evident that the number of blood samples collected in the clinical pharmacology study is as critical as the number of patients available and the dose levels under consideration for the study.^18^, ^50^ Tools have been developed in statistical research to provide insight into the contribution of (individual) input data to the overall precision of parameter estimates.^51^ These techniques can become powerful when combined with new sampling techniques such as dried blood spots or microsampling, particularly in special paediatric patient groups such as neonates. Clinical study simulations can be further implemented to illustrate the impact of different sampling and design scenarios, thereby justifying the proposed sampling scheme and overall protocol design. On the other hand, one should also consider that additional sampling for drug or metabolite may be required if more than efficacy is to be established. Opportunistic (ad hoc) sampling should be considered when acute adverse events occur.

One last keynote on the advantages of PKPD studies is the possibility of establishing the clinical relevance of covariate factors known to affect pharmacokinetics or pharmacodynamics in children. Therefore, attention must be given to the way information is collected in these kinds of trials, especially the so‐called time‐varying covariates, such as age, body weight, BSA and many biochemical and haematological parameters (clinical laboratories) which may be closely linked to organ function and reflect differences in drug disposition or pharmacodynamics. In addition, information regarding the onset of disease, phenotype, genotype, time since diagnosis, concomitant and recent drug therapy should also be considered as relevant factors in some diseases. It should be noted that some covariate factors will be relevant only in a subgroup of patients, for example organ maturation, whereas others can affect the whole patient population.

## Conclusions

Paediatricians can and should perform investigator‐initiated clinical pharmacological research in children as there are many gaps in the knowledge about drugs used for children. To develop rational, patient‐tailored dosing schemes, population PKPD studies in children and infants are needed. The emergence of new laboratory techniques and statistical tools allows for the analysis of sparse and unbalanced data and has increased the possibilities to perform (observational) PKPD studies in the paediatric clinic. To improve the quality of future paediatric PKPD investigations, and to prevent the conduct of paediatric RCTs that are doomed to fail because of inadequate dosing, the experience and knowledge about these tools is shared in this study. If performed well, the results of these studies will contribute to the evidence base underlying clinical guidelines and regulatory decisions concerning labelling adjustments.

In contrast to the design of RCTs for the assessment of efficacy, in which the aim is to minimise the signal‐to‐noise ratio, studies aimed at the characterisation of the exposure–response curve and subsequent dose selection of a drug need to consider the sources of variation in the target population. This means that in the design of a paediatric PKPD study, intrinsic factors determining variability in drug exposure and response, such as age, weight and gender, will have to be accounted for carefully to maximise the amount of information gathered from the smallest possible number of participating children.

## Declarations

## References

[1] Cella M *et al* What is the right dose for children? Br J Clin Pharmacol 2010; 70: 597–603. 21087295 10.1111/j.1365-2125.2009.03591.x PMC2950994

[2] European Commission . Better medicines for children. From concept to reality. Progress report on the paediatric regulation (EC) No 1901/2006. Brussels: European Commission, 2013.

[3] European Medicines Agency . Paediatric investigation plans: templates, forms and submission dates. European Medicines Agency, 2016 Available from http://www.ema.europa.eu/ema/index.jsp?curl=pages/regulation/document_listing/document_listing_000293.jsp

[4] Laughon MM *et al* Drug labeling and exposure in neonates. JAMA Pediatr 2014; 168: 130–136. 24322269 10.1001/jamapediatrics.2013.4208 PMC3927948

[5] Haslund‐Krog S *et al* The impact of legislation on drug substances used off‐label in paediatric wards – a nationwide study. Eur J Clin Pharmacol 2014; 70: 445–452. 24398969 10.1007/s00228-013-1626-1

[6] European Medicines Agency . Paediatric needs, 2013 Available from: http://www.ema.europa.eu/ema/index.jsp?curl=pages/regulation/document_listing/document_listing_000096.jsp.

[7] Starkey ES , Sammons HM . Practical pharmacokinetics: what do you really need to know? Arch Dis Child Educ Pract Ed 2015; 100: 37–43. 25122157 10.1136/archdischild-2013-304555

[8] Turner MA *et al* Paediatric drug development: the impact of evolving regulations. Adv Drug Deliv Rev 2014; 73: 2–13. 24556465 10.1016/j.addr.2014.02.003

[9] Wimmer S *et al* The EU paediatric regulation: still a large discrepancy between therapeutic needs and approved paediatric investigation plans. Paediatr Drugs 2014; 16: 397–406. 25056717 10.1007/s40272-014-0082-4

[10] European Union . Clinical trials: clearer rules, better protection, 2015 Available from: http://www.europarl.europa.eu/news/en/news-room/content/20130527IPR10525/html/Clinical-trials-clearer-rules-better-protection.

[11] EMA . Regulation, 2015 Available from: http://www.ema.europa.eu/ema/index.jsp?curl=pages/regulation/document_listing/document_listing_000122.jsp&mid=WC0b01ac0580022bb2#section2

[12] EMA . Enprema, 2015 Available from: http://www.ema.europa.eu/ema/index.jsp?curl=pages/partners_and_networks/general/general_content_000303.jsp.

[13] Connor EM *et al* Meeting the demand for pediatric clinical trials. Sci Transl Med 2014; 6: 227 fs11. 10.1126/scitranslmed.3008043 PMC4104358 24622511

[14] Rieder M , Hawcutt D . Design and conduct of early phase drug studies in children: challenges and opportunities. Br J Clin Pharmacol 2016; doi: 10.1111/bcp.13058 [Epub ahead of print]. PMC5061783 27353241

[15] Benjamin DK Jr *et al* Pediatric antihypertensive trial failures: analysis of end points and dose range. Hypertension 2008; 51: 834–840. 18332283 10.1161/HYPERTENSIONAHA.107.108886 PMC2782749

[16] American Academy of Pediatrics . Policy Statement. Off‐Label Use of Drugs in Children. American Academy of Pediatrics, 2014; 133: 563–567.

[17] Zhao W *et al* Dosage individualization in children: integration of pharmacometrics in clinical practice. World J Pediatr 2014; 10: 197–203. 25124969 10.1007/s12519-014-0493-x

[18] Viele K , Connor JT . Dose‐finding trials: optimizing phase 2 data in the drug development process. JAMA 2015; 314: 2294–2295. 26624828 10.1001/jama.2015.16702

[19] Bellanti F , Della Pasqua O . Modelling and simulation as research tools in paediatric drug development. Eur J Clin Pharmacol 2011; 67(Suppl 1): 75–86. 21246352 10.1007/s00228-010-0974-3 PMC3082698

[20] Bellanti F *et al* Integration of PKPD relationships into benefit‐risk analysis. Br J Clin Pharmacol 2015; 80: 979–991. 25940398 10.1111/bcp.12674 PMC4631171

[21] Cella M *et al* Bridging strategies for drug combinations in pediatric indications. Clin Pharmacol Ther 2012; 91: 726–733. 22398968 10.1038/clpt.2011.298

[22] Piana C *et al* Influence of covariate distribution on the predictive performance of pharmacokinetic models in paediatric research. Br J Clin Pharmacol 2014; 78: 145–157. 24433411 10.1111/bcp.12322 PMC4168389

[23] Piana C *et al* A model‐based approach for the evaluation of once daily dosing of lamivudine in HIV‐infected children. Br J Clin Pharmacol 2014; 77: 852–860. 24118047 10.1111/bcp.12246 PMC4004405

[24] Dunne J *et al* Extrapolation of adult data and other data in pediatric drug‐development programs. Pediatrics 2011; 128: e1242–e1249. 22025597 10.1542/peds.2010-3487

[25] European Medicines Agency . Concept paper on extrapolation of efficacy and safety in medicine development, Draft. European Medicines Agency, 2012 Available from: http://www.ema.europa.eu/docs/en_GB/document_library/Scientific_guideline/2012/06/WC500129285.pdf.

[26] European Medicines Agency . Reflection paper on extrapolation of efficacy and safety in paediatric medicine development, Draft. European Medicines Agency, 2016 Available from: http://www.ema.europa.eu/docs/en_GB/document_library/Regulatory_and_procedural_guideline/2016/04/WC500204187.pdf.

[27] Madabushi R *et al* Pharmacokinetic and pharmacodynamic basis for effective argatroban dosing in pediatrics. J Clin Pharmacol 2011; 51: 19–28. 20421511 10.1177/0091270010365550

[28] De Cock RF *et al* The role of population PK‐PD modelling in paediatric clinical research. Eur J Clin Pharmacol 2011; 67(Suppl 1): 5–16. 20340012 10.1007/s00228-009-0782-9 PMC3082690

[29] van den Anker JN *et al* Developmental pharmacokinetics. Handb Exp Pharmacol 2011; 205: 51–75. 21882105 10.1007/978-3-642-20195-0_2

[30] Allegaert K , van den Anker JN . Clinical pharmacology in neonates: small size, huge variability. Neonatology 2014; 105: 344–349. 24931327 10.1159/000360648 PMC4111147

[31] Centers for Disease Control and Prevention . Growth charts, 2016 Available from: https://www.cdc.gov/growthcharts/.

[32] Cella M *et al* Adaptive trials in paediatric development: dealing with heterogeneity and uncertainty in pharmacokinetic differences in children. Br J Clin Pharmacol 2012; 74: 346–353. 22256787 10.1111/j.1365-2125.2012.04187.x PMC3630754

[33] Hampson LV *et al* Bridging the gap: a review of dose investigations in paediatric investigation plans. Br J Clin Pharmacol 2014; 78: 898–907. 24720849 10.1111/bcp.12402 PMC4239983

[34] Bellanti F *et al* Sampling optimisation in pharmacokinetic bridging studies: example on the use of deferiprone in children with beta‐thalassaemia. J Clin Pharmacol 2016; 56: 1094–1103. 26785826 10.1002/jcph.708

[35] Wang JJ , Ivanova A . Dose finding with the sequential parallel comparison design. J Biopharm Stat 2014; 24: 1091–1101. 24919070 10.1080/10543406.2014.924960

[36] Desfrere L *et al* Dose‐finding study of ibuprofen in patent ductus arteriosus using the continual reassessment method. J Clin Pharm Ther 2005; 30: 121–132. 15811164 10.1111/j.1365-2710.2005.00630.x

[37] Treluyer JM *et al* Minimum effective dose of midazolam for sedation of mechanically ventilated neonates. J Clin Pharm Ther 2005; 30: 479–485. 16164495 10.1111/j.1365-2710.2005.00678.x

[38] Santen G *et al* From trial and error to trial simulation. Part 1: the importance of model‐based drug development for antidepressant drugs. Clin Pharmacol Ther 2009; 86: 248–254. 19657333 10.1038/clpt.2009.105

[39] Santen G *et al* From trial and error to trial simulation. Part 2: an appraisal of current beliefs in the design and analysis of clinical trials for antidepressant drugs. Clin Pharmacol Ther 2009; 86: 255–262. 19675543 10.1038/clpt.2009.107

[40] Frank LM *et al* Lamictal (lamotrigine) monotherapy for typical absence seizures in children. Epilepsia 1999; 40: 973–979. 10403222 10.1111/j.1528-1157.1999.tb00805.x

[41] Avramis VI , Spence SA . Clinical pharmacology of asparaginases in the United States: asparaginase population pharmacokinetic and pharmacodynamic (PK‐PD) models (NONMEM) in adult and pediatric ALL patients. J Pediatr Hematol Oncol 2007; 29: 239–247. 17414566 10.1097/MPH.0b013e318047b79d

[42] Brussee JM *et al* Children in clinical trials: towards evidence‐based pediatric pharmacotherapy using pharmacokinetic‐pharmacodynamic modeling. Expert Rev Clin Pharmacol 2016; 9: 1235–1244. 27269200 10.1080/17512433.2016.1198256

[43] Krekels EH *et al* Pediatric pharmacology: current efforts and future goals to improve clinical practice. Expert Opin Drug Metab Toxicol 2015; 11: 1679–1682. 26166262 10.1517/17425255.2015.1065815

[44] Nyberg J *et al* Methods and software tools for design evaluation in population pharmacokinetics‐pharmacodynamics studies. Br J Clin Pharmacol 2015; 79: 6–17. 24548174 10.1111/bcp.12352 PMC4294071

[45] Dartois C *et al* Overview of model‐building strategies in population PK/PD analyses: 2002‐2004 literature survey. Br J Clin Pharmacol 2007; 64: 603–612. 17711538 10.1111/j.1365-2125.2007.02975.x PMC2203272

[46] Norman GR *et al* Methodological problems in the retrospective computation of responsiveness to change: the lesson of Cronbach. J Clin Epidemiol 1997; 50: 869–879. 9291871 10.1016/s0895-4356(97)00097-8

[47] Sheiner LB . Learning versus confirming in clinical drug development. Clin Pharmacol Ther 1997; 61: 275–291. 9084453 10.1016/S0009-9236(97)90160-0

[48] GRIP . Educational programme, 2016 Available from: http://www.gripnetwork.org/index.php/cms/en/training_and_education.

[49] Wang Y *et al* Clarification on precision criteria to derive sample size when designing pediatric pharmacokinetic studies. J Clin Pharmacol 2012; 52: 1601–1606. 22162537 10.1177/0091270011422812

[50] Roberts JK *et al* Optimal design in pediatric pharmacokinetic and pharmacodynamic clinical studies. Paediatr Anaesth 2015; 25: 222–230. 25580772 10.1111/pan.12575

[51] Bijleveld Y *et al* A simple quantitative method analysing amikacin, gentamicin, and vancomycin levels in human newborn plasma using ion‐pair liquid chromatography/tandem mass spectrometry and its applicability to a clinical study. J Chromatogr B Analyt Technol Biomed Life Sci 2014; 951–952: 110–118. 10.1016/j.jchromb.2014.01.035 24548921

